# Protein dynamics and the allosteric transitions of pentameric receptor channels

**DOI:** 10.1007/s12551-014-0149-z

**Published:** 2014-11-29

**Authors:** Jean-Pierre Changeux

**Affiliations:** 1 UMR 3571 CNRS, Institut Pasteur, 25 rue du Docteur Roux, 75015 Paris, France; 2 Collège de France, 11 Place Marcelin Berthelot, 75005 Paris, France; 3 Kavli Brain-Mind Institute University of California, San Diego, CA USA

**Keywords:** Allosteric interactions, Acetylcholine receptor, Molecular dynamics, Conformational changes

## Abstract

The recent application of molecular dynamics (MD) methodology to investigate the allosteric transitions of the acetylcholine receptor and its prokaryotic and eukaryotic pentameric homologs has yielded new insights into the mechanisms of signal transduction by these receptors. Combined with available data on X-ray structures, MD techniques enable description of the dynamics of the conformational change at the atomic level, intra-molecular propagation of this signal transduction mechanism as a concerted stepwise process at physiological timescales and the control of this process by allosteric modulators, thereby offering new perspectives for drug design.

## Definition of allosteric interactions: importance of molecular dynamics

We have recently celebrated the “50th anniversary of allostery”, and the concept is alive and well. Allosteric interactions between proteins and their regulatory ligands were initially defined as indirect interactions between topographically distinct sites that are mediated by a discrete reversible alteration of the molecular structure of the protein—and this definition is still valid (for review, Changeux 2013a). The concept was first proposed in 1961 to account for the feedback inhibitory mechanism mediated by the first enzyme of bacterial biosynthetic pathways, in which the feedback inhibitor is not a steric analog of the substrate (Changeux 1961; Monod and Jacob 1961; Gerhart and Pardee 1962). It was expanded in 1963 (Monod et al. 1963) to explain the properties of regulatory proteins in general, including Perutz’s structural data on hemoglobin, in the framework of Koshland’s (1959) “induced fit” mechanism, according to which the ligand “instructs” rather than “selects” the protein conformational change.

In 1965, attention focused on the observation that, in many regulatory proteins, in addition to, and possibly as part of, the signal transduction mechanism, substrates and regulatory ligands interact in a cooperative manner. It was also noted that all of these interactions may be simultaneously uncoupled by a variety of chemical or physical treatments (Changeux 1961; Gerhart and Pardee 1962). These observations point to a global mechanism at the protein level that makes these regulatory enzymes function as “molecular switches” (Monod et al. 1965; for review, Changeux 2012a, 2013a,b).

To explain the particular protein design involved, a structural hypothesis was proposed where the cooperativity observed between the multiple binding sites for the substrate and regulatory ligand relies on the cooperative organization of the protein into “oligomers” comprising a small number of repeated units and possessing at least one axis of symmetry (Monod et al. 1965). A second critical assumption was that, unlike in the induced-fit model, the regulatory oligomers naturally exist *in the absence of the ligand* in at least two discrete conformations, the R state (for relaxed) and the T state (for constrained), which are in thermodynamic equilibrium. These states differ, in particular, in their tertiary distribution and/or inter-subunit bond energy (quaternary constraint). Ligands would then shift the conformational equilibrium by stabilizing the oligomer conformation for which they have the highest affinity, thus mediating signal transduction. Substrate and activators would stabilize the R state, inhibitors the T state. The model, termed the Monod–Wyman–Changeux (MWC) model (Monod et al. 1965), assumed that the conformational transition occurs simultaneously for all subunits: it is “concerted” and conserves the oligomer symmetry. Shortly thereafter, in 1966, Koshland et al. proposed a sequential induced-fit mechanism of allosteric transition, referred to as the Koshland–Nemethy–Filmer (KNF) model, which involved a progressive conformational change with ligand binding that excludes any conformational change of the protein in the absence of the ligand.

Abundant studies carried out with a large diversity of regulatory proteins, including neurotransmitter receptors (Changeux 2013a), have lent support to, and further extended, the MWC model, emphasizing in particular “population shifts” within the energy landscape formalism (see Cui and Karplus 2008 ; Itoh and Sasai 2010; Changeux 2012a, 2013a; Terada et al. 2013; Motlagh et al. 2014; Tsai 2014). Importantly, both the MWC and KNF models formulate a *static* (end-point) equilibrium picture of the allosteric transition. To achieve progress toward the *dynamic* nature of the phenomenon, complementary and time-resolved analyses, such as molecular dynamics and novel technologies, were needed. As stated by Cui and Karplus (2008), inclusion of atomic fluctuations opens the way to a more sophisticated and accurate interpretation of protein activity that is essential for understanding the mechanism of allosteric interactions. In this review, I shall examine this issue with the nicotinic acetylcholine receptor (nAChR), a neurotransmitter-gated ion channel, which has served in past decades as a privileged model of regulatory protein engaged in intercellular communication in the nervous system. On the basis of recently available crystallographic data, molecular dynamics models of the signal transduction process they mediate have been elaborated and compared with the in vivo physiological data.

## The concept of pharmacological receptor, the identification of the nicotinic receptor and the ionic response to acetylcholine

Ever since Claude Bernard’s pioneering work on the effect of curare, the chemistry of intercellular communications has relied on the concept of the pharmacological “receptor” laid down by the English pharmacologist John Newport Langley (1905). It took however 65 years to chemically identify—through research involving the fish electric organ and a snake venom toxin—the first neurotransmitter receptor, the nicotinic receptor (nAChR) from the neuromuscular junction (Changeux et al. 1970; Miledi et al. 1971 ; Karlin 1993; for review, Changeux 2012b). nAChRs are involved in many brain processes and diseases, such as attention, learning and memory, access to consciousness, nicotine addiction and Alzheimer and Parkinson diseases (for review, Changeux 2006, 2010). Understanding the functional organization and dynamics of these receptors at the atomic level is thus of considerable interest, both in itself and for the development of new therapeutics.

**Fig. 1:**
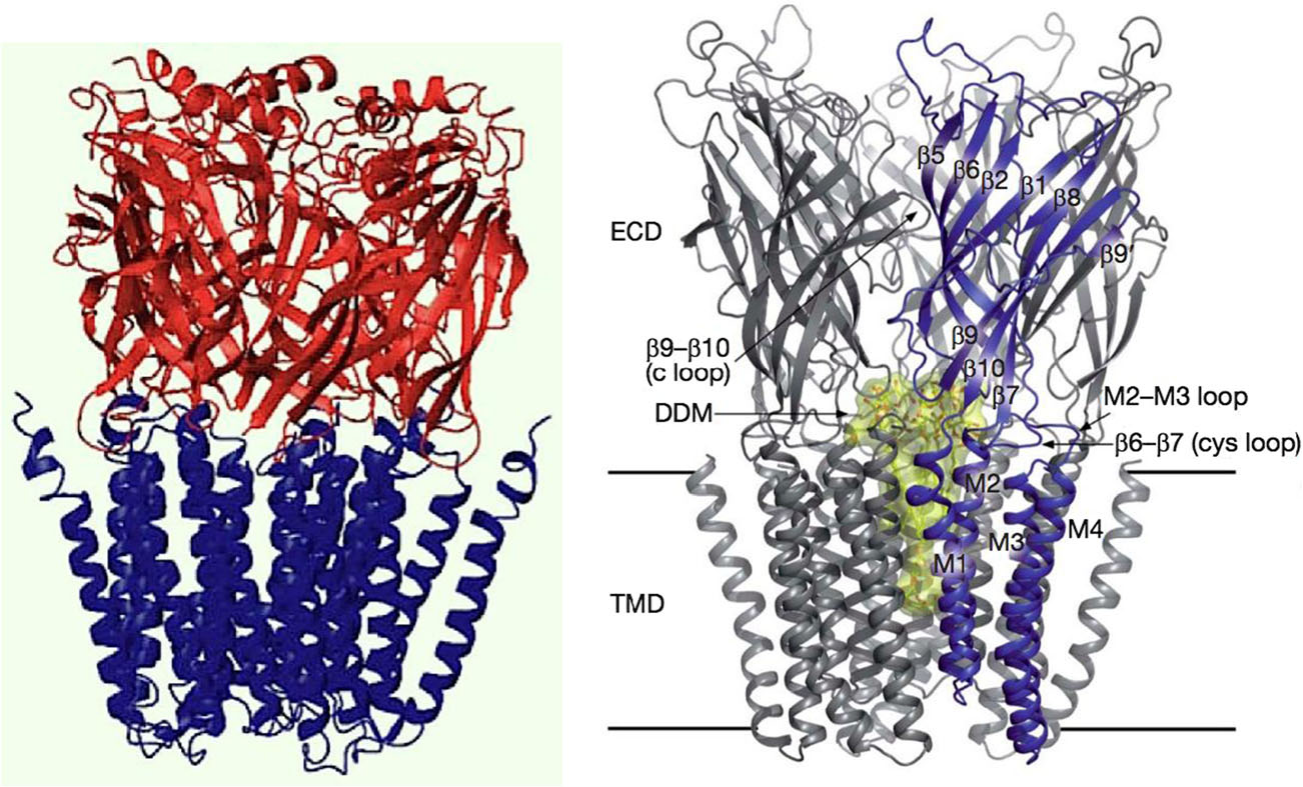
*Left *Model of the α7 nicotinic acetylcholine receptor (nAChR) elaborated by Taly et al. (2005) from the X-ray crystal structure of the snail acetylcholine binding protein, a homolog of the extracellular domain (Brejc et al. 2001) and the lower resolution cryo-electron microscopy data of *Torpedo* nAChR (Unwin 2005) for the membrane domain. From Taly et al. (2005).* Right* Crystal structure of a prokaryotic homolog of the nAChR from *Gloeobacter violaceus* [*G. violaceus* ligand-gated ion channel (GLIC)] in its open-channel conformation (Bocquet et al. 2009).* ECD* Extracellular domain,* TMD* transmembrane domain of four transmembrane α-helices (*M1*–*M4*) per subunit, DDM detergent dodecyl maltoside-blocked ion channel (*yellow*). The homology between eukaryotic and prokaryotic receptors is remarkable. From Bocquet et al (2009)

nAChRs are integral allosteric membrane proteins with a molecular mass of approximately 290 kDa that form oligomers comprising five identical or homologous subunits symmetrically arranged around a central ion channel, with a fivefold symmetry axis perpendicular to the membrane (for review, Changeux and Edelstein 2005) (Fig. 1).The primary structure of each subunit consists of a large hydrophilic amino-terminal extracellular (EC) domain, a transmembrane (TM) domain comprising four hydrophobic segments (M1–M4) and a variable hydrophilic cytoplasmic or intracellular domain. There are two to five ACh binding sites within the EC domain located at the boundary between subunits. These ACh binding sites are far apart (approx. 60 Å) but still functionally linked to a single cationic ion channel located on the axis of symmetry of the TM domain and delineated by the M2 α-helix (Fig. 1). The interaction between neurotransmitter site and ion channel is thus typically “allosteric.” The EC and TM additionally carry several allosteric modulatory sites for natural (e.g. Ca^++^, lipids) and synthetic (e.g. ivermectin) ligands. Therefore, nAChRs possess the structural elements required to convert a chemical signal, typically a local increase in extracellular ACh concentration, into an electrical signal generated by the opening of the ion channel. Over the years the nAChR has become the “founding father” of the broader superfamily of pentameric receptors which includes the 5-hydroxytryptamine receptor (5HT3R), the inhibitory anion-selective γ-aminobutyric acid type A (GABA_A_) and glycine receptors and the invertebrate glutamate-gated chloride channel (GluCl) (Changeux 2012b).

An important outcome of Langley’s receptor theory (1905) was the development of electrophysiological recordings aimed at understanding the ionic response of receptors to neurotransmitters (Katz 1966) with the important addition of the time dimension missing in the biochemical–structural approach at the time. At the neuromuscular junction, the postsynaptic potential elicited by either electrical stimulation of the nerve ending or iontophoretic application of ACh has a fast (<0.2 ms) signal rise time and a signal decay time of a few milliseconds. The local concentration of ACh transiently rises (<1 ms) to 3 × 10^−4^ M over a background release of 10^−8^ M (Katz and Miledi 1973, 1977; Kuffler and Yoshikami 1975). These data were interpreted in terms of the then available models of enzyme kinetics, in particular acetylcholine esterase (Augustinsson 1948, 1950), assuming a two-step process which consisted of the formation of the receptor–ACh complex followed by opening of the channel (Del Castillo and Katz 1957). This model was consistent with Koshland’s induced-fit mechanism (1959):

$$ \mathrm{R} + \mathrm{A} < - - > \mathrm{R}\mathrm{A} < - - > \mathrm{R}*\mathrm{A}\left(\mathrm{open}\right). $$

The application of patch-clamp recording techniques revealed that the cellular postsynaptic response can be reduced to the collective opening of separate molecular channels, with each individual opening having a square shape with a rise time in the microsecond range and a mean open time of a few milliseconds (Neher and Sakmann 1976). These values set the time-range for ongoing molecular dynamics studies (see below). Langley (1905) had already noticed that prolonged application of the agonist nicotine blocks receptor responses, resulting in desensitization of the receptor. To fit the electrophysiological data then available, Katz and Thesleff (1957) proposed that ACh slowly (on a 10 ms to 1 s timescale) stabilizes a new high-affinity closed (“refractory”) state of the receptor which, unlike the “effective” excitable state, would pre-exist ligand binding. Subsequent electrophysiological and biochemical studies with nAChR-rich “excitable” membrane fragments (Kasai and Changeux 1971; Cohen et al. 1972) and a fluorescent analog of acetylcholine (dansyl-C6-choline) enabled researchers to follow directly in vitro the binding kinetics of a nicotinic ligand and its conformational and ionic consequences without using in vivo electrophysiological recordings (Heidmann and Changeux 1979a, b, 1980; Heidmann et al. 1980, see also Neubig and Cohen 1980; Neubig et al. 1982 with radioactive ligands). Extensive kinetic analyses with an adequate rapid mixing apparatus (2.5-ms dead time) resulted in the first experimental in vitro demonstration in the millisecond range of the allosteric transitions of the receptor protein’s multiple conformational states: (1) a resting closed-channel R state stabilized by nicotinic antagonists; (2) an active, fast, open-channel A state with a low affinity for ACh and nicotinic agonists (kDa ACh: approx.50–100 μM); (3) at least a fast I and a slow D desensitized, refractory state, with higher affinities for agonists (but also for antagonists) [(kDa of I for ACh: approx.1 μM; kDa of D for ACh: approx. 3–5 nM] (Heidmann and Changeux 1980; see Edelstein et al. 1996). In contrast to a widespread opinion among pharmacologists, the highest affinity states do not correspond to the active functional state of the receptor—quite the contrary is true.

The scheme presented below (Changeux 1990) illustrates the interconversion between the four different states. These states are postulated to occur spontaneously in the absence of ligands. Orthosteric and allosteric (see chapter 6 from Changeux 1990) ligands, binding differentially and selectively, stabilize the state(s) to which each ligand interacts with the highest affinity and, consequently, in the case of agonists (here ACh), mediate signal tranduction. Competitive blockers (CB) channel blockers (NCB) and/or allosteric modulators (AM) may selectively stabilize any of the states at the level of sites distinct from the ACh binding site and ion channel (see chapter 6 from Changeux 1990).
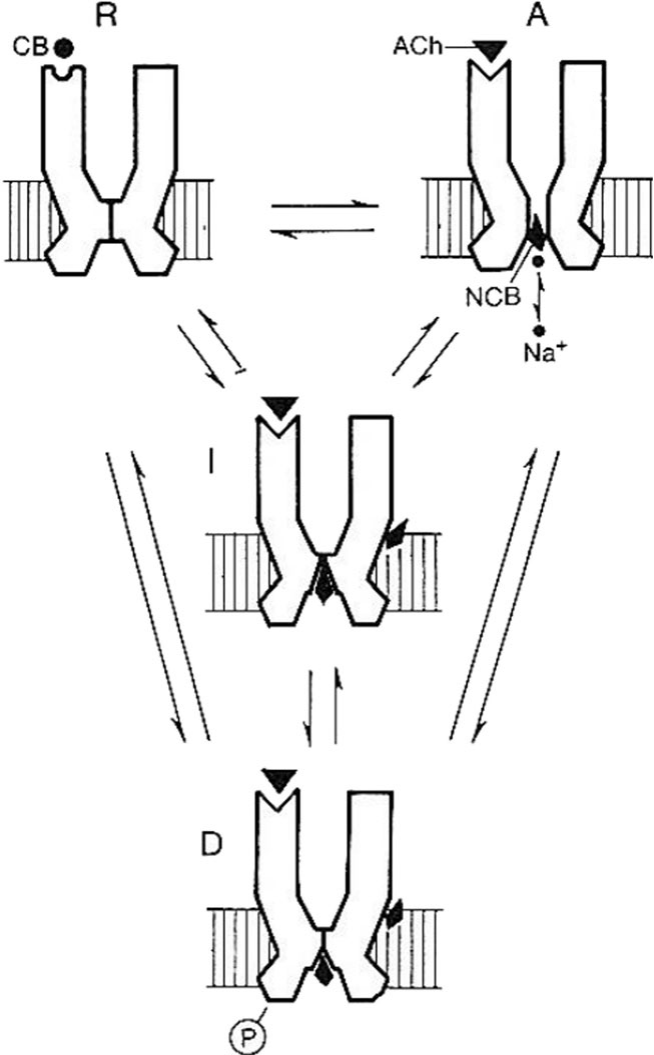


Moreover, a non-negligible fraction (approx. 20 %) of the receptor resides in the high-affinity D state in the total absence of ligand (Heidmann and Changeux 1979a, b, 1980). In parallel studies, Jackson (1984) observed the spontaneous opening of muscle nAChR in the absence of ACh. Purohit and Auerbach (2013) have recently extended the analysis and elegantly demonstrated that “diliganded and brief unliganded openings are generated by the same essential, global transition”, thereby ruling out the induced-fit mechanism and validating the fundamental premises of the MWC model that the basic activation mechanism involves a unique R <−−> A conformational transition that is independent of ligand binding (Auerbach 2012, 2014).

## Crystallographic structure of prokaryotic and eukaryotic pentameric receptors

An important step in the development of the molecular dynamics analysis of pentameric receptors was the availability of high-resolution structural data. Early electron micrographs of nAChR from fish electric organs (Cartaud et al. 1973; Brisson and Unwin 1985a, b) together with the X-ray structure of the homopentameric, water-soluble acetylcholine binding protein (AChBP) from *Lymnaea stagnalis* (Brejc et al. 2001), which displays significant (approx. 30 %) sequence homology with the EC domain of the nAChRs and remarkable conservation of the binding site residues, led to the development of atomic models of the full-length nAChR (Unwin 2005, 2013) (see Taly et al. 2014 for discussion). However, the resolution remained low (>4 Å) due to sample preparation conditions which unfortunately were known to “uncouple” receptor function (Paas et al. 2005; daCosta et al. 2013).

The situation changed dramatically with the discovery in bacteria of DNA sequences homologous to eukaryotic nAChR (Tasneem et al. 2005) and the subsequent cloning and expression in eukaryotic cells of one of these sequences from the photosynthetic bacterium* Gloeobacter violaceus* (Bocquet et al. 2007). The protein was demonstrated by electrophysiological recordings to behave as a ligand-gated ion channel activated at acidic pHs (Bocquet et al. 2007). Purification and crystallization of the *G. violaceus* ligand-gated ion channel (GLIC) and of a closely related protein led to the resolution of the first X-ray structure of a pentameric ligand-gated ion channel (pLGIC) in a closed-channel state (resolution 3.3 Å) from *Erwinia chrysanthemi* (ELIC) (Hilf and Dutzler 2008) and in an open-channel conformation (resolution 2.9 Å) from *G. violaceus* (GLIC) (Bocquet et al. 2009; Hilf and Dutzler 2009). The structure of an eukaryotic member of the family, the anionic glutamate receptor from *Caenorhabditis elegans* (GluCl), was then solved in an open conformation as a complex with the positive allosteric modulator ivermectin, revealing an astonishing structural similarity with the three-dimensional (3D) structure of GLIC (Hibbs and Gouaux 2011). The recent crystallographic structures of the eukaryotic GABA_A_ receptor (Miller and Aricescu 2014) and 5HT_3_ receptor (Hassaine et al. 2014) confirm a common structural organization of the constituent subunits. The EC domain folds into a highly conserved immunoglobulin-like β-sandwich and the TM domain consists of four α-helices organized as a well-conserved bundle [in agreement with the low-resolution electron microscopy structures of *Torpedo * nAChR (Unwin 2005)]. The M2 helix lines the channel walls (Giraudat et al. 1986, 1987; Hucho et al. 1986; Imoto et al. 1986, 1988) and is surrounded by a ring of α-helices made of M1 and M3. The fourth TM α-helix, M4, is the most peripheral helix and interacts extensively with the lipid bilayer (Bocquet et al. 2009). Also, the cytoplasmic domain, absent in prokaryotic receptors, was revealed for the first time in 5HT3 receptors (Hassaine et al. 2014), further extending our structural knowledge of the family. The access to high-resolution full X-ray structures of pentameric receptors legitimated a reliable analysis of the dynamics of their conformational change.

## Early studies on the molecular dynamics of signal tranduction mediated by pentameric receptors

Even before high-resolution structural data became accessible, Taly et al. (2005) developed, by comparative analysis, a 3D model of the α7-nAChR on which they performed the first, coarse-grained, molecular dynamics simulation of a pLGIC using the method referred to as “normal mode analysis.” By approximating the surface of the conformational landscape, the analysis decomposed the receptor protein movements into discrete modes. Among the ten modes with the lowest frequency, the first showed a structural reorganization described as a concerted rotation, in opposite directions, of the upper EC and lower TM domains around the pore axis—a movement referred to as a *quaternary twist*. The twist affects the structure of the ion channel toward its opening and results in a significant reshaping of the subunits’ interfaces which open and close the agonist binding site(s) located at these interfaces. These observations were confirmed and extended with another model of α7-nAChR (Cheng et al. 2006), with the crystal structures of GLIC (Bocquet et al. 2009) and of ELIC (Cheng et al. 2009) and with a 1-μs-long all-atom molecular dynamics simulation of GLIC (Nury et al. 2010). In parallel, another computational study (Taly et al. 2006) on nAChR was undertaken, involving pathological mutations associated with congenital myasthenia and autosomal dominant nocturnal frontal lobe epilepsy. These mutations constitutively stabilize the receptor in an active open conformation, even in the absence of agonist. The substituted amino acids were found to be located at interfaces either between subunits or, within a given subunit, between rigid blocks and thus to alter the twisting mode. Taken together, these results support the conclusion that quaternary twisting is a robust structural motion that accompanies the opening of the ion channel and possibly other moves of the channel, such as those occurring during desensitization.

In parallel electrophysiological studies, Auerbach and colleagues (Grosman et al. 2000; Purohit et al. 2007, 2013; Purohit and Auerbach 2013) examined the gating dynamics of muscle nAChR closed–open transition state ensembles (TSE). These authors measured the single-channel opening (*k*_o_) and closing (*k*_c_) rate constants of sets of receptor mutants with various side chain substitutions for individual amino acids. For each series of mutants, properties of the TSE could be deduced from ϕ (phi), the slope of a log–log plot of* k*_o_ versus* K*_eq_ (*k*_o_/*k*_c_). The analysis suggests that the overall nAChR isomerization consists of a well-defined sequence of protein domain motions that generate a propagated, Brownian stepwise process. Two separate regions in the α-subunits, namely, transmitter-binding sites and linkers in the membrane domain, have the highest ϕ values (i.e. change conformation the earliest), followed by the EC domain, then most of the membrane domain and finally the gate, resulting in channel opening (Purohit et al. 2013). Accordingly, the gating dynamics of muscle nAChR does not proceed as a single-step “rigid” event but through a concerted, step-wise, conformational mechanism that can be investigated by structural analysis and molecular dynamics simulations.

## Structural dynamics model of signal tranduction: an emerging model.

On the basis of the GLIC open state, the ELIC “undefined” closed state and the GluCl open-channel structure (when bound to the positive allosteric modulator ivermectin), Calimet et al. (2013) carried out extensive all-atom molecular dynamics simulations in the timescale of 0.2–0.5 μs. Upon removal of ivermectin, the simulated trajectory of GluCl reveals a stepwise sequence of structural events that couple agonist unbinding from the EC domain to ion-pore closing in the TM domain. The simulation also shows that, in agreement with Taly et al. (2005), a global twisting initiates the closing transition from the open state by facilitating the (un)-tilting of the pore-lining helices. The mechanistic scenario emerging from the simulations suggested that receptor twisting contributes to the activation process by “locking” the ion channel in the open-pore form. In addition, a large reorientation or *tilting* of the extracellular β-sandwiches in the outward direction further contributes to the allosteric communication between the neurotransmitter-binding site and the ion pore.

**Fig. 2:**
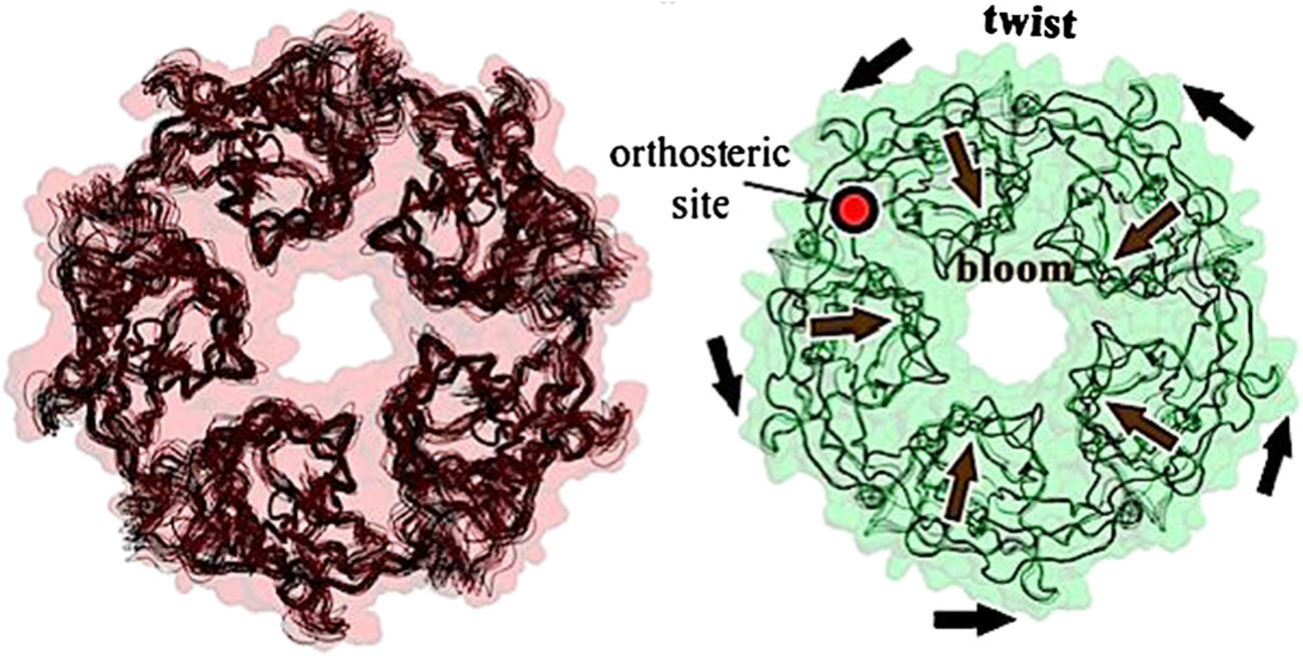
Compared crystal structures of the closed (pH 7; *left*) and open (pH 4; *right*) channel states of the receptor from *G. violaceus* (GLIC). The pH 7 state shows a structural variability which is absent in the pH 4 state.* Arrows* in the *right structure* illustrate the concerted quaternary motions of twist and bloom occurring during signal transduction. From Sauguet et al. 2014a, Fig. 4A.

**Fig. 3:**
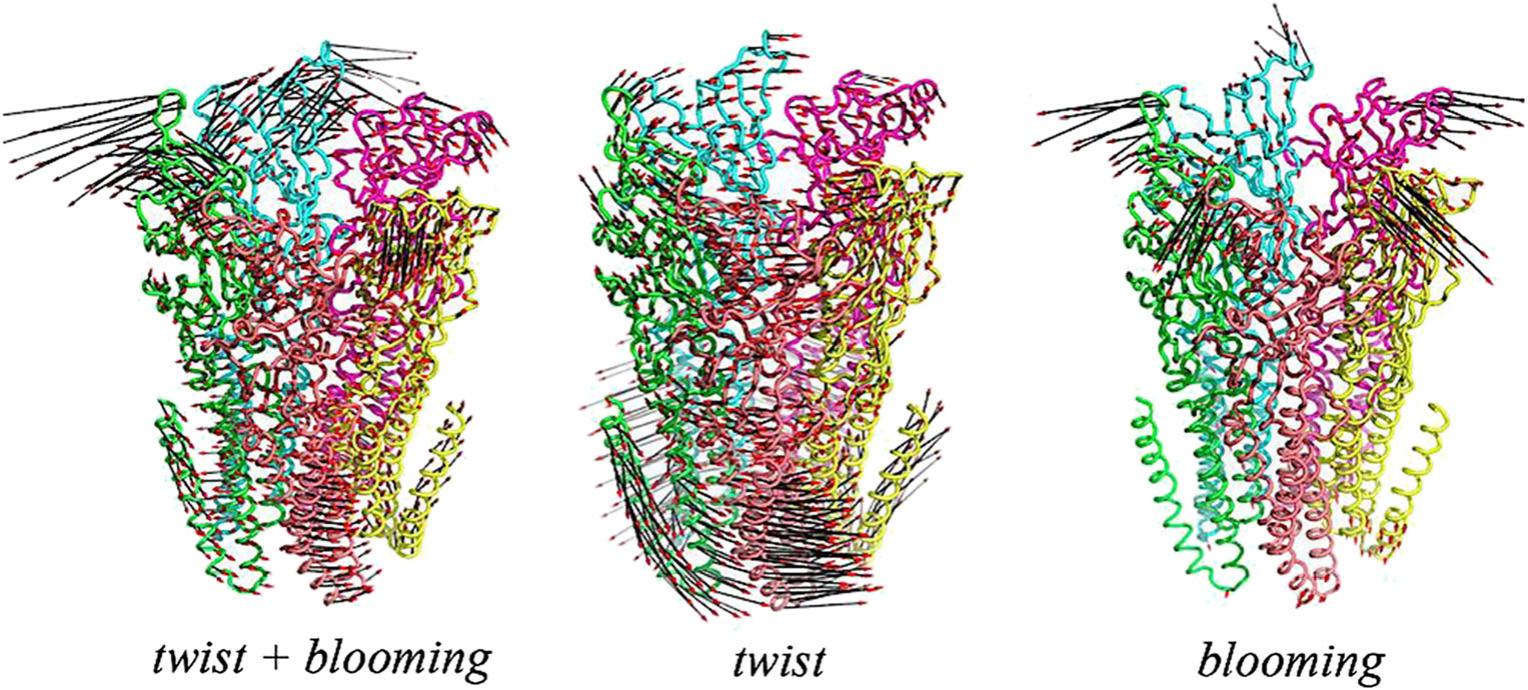
Analysis of the quaternary motions of the GLIC* twist* and* blooming* involved in the allosteric transition linking the ECD and the ion channel. Comparing the resulting profiles between the pH 7 and pH 4 structures highlights the combination of radial and tangential movements that relate them in the course of channel opening and are viewed as corresponding to two different normal modes: twist and blooming, respectively.* Difference vectors* pH 7 (closed channel) to pH 4 (open channel). From *left* to *right* Twist and blooming; twist; blooming. From Sauguet et al. 2014a, Figure S8b

The crystal structures of GLIC at both pH 7 (closed) and pH 4 (open) further provided, for the first time, the end-point structures of the gating mechanism in the same pLGIC (Sauguet et al. 2014a) and included a coarse-grained dynamic modeling of the structures (Sauguet et al. 2014a) (Fig. 2). These data revealed the occurrence of a large *quaternary twisting* upon receptor activation, as well as the occurrence of important tertiary changes on activation, in particular a significant *tilting* of the M2 helices. Remarkably, the X-ray structures show, in agreement with Calimet et al. (2013), a radial expansion of the EC domain, termed *outward tilting* or *blooming*, which reflects a reorientation of the β-sandwiches (Fig. 3).

The recent data on GluCl structure in its *apo* resting conformation compared to that obtained in the presence of the positive allosteric modulator ivermectin appears, to some extent, to be consistent with some of the conformational changes identified with GLIC (Sauguet et al. 2014a): a *twist* of the EC domain and a quaternary un-*blooming* (“resembling the closure of a blossom”) occuring upon activation (Althoff et al. 2014). However,in the GluCl structure, functionally relevant tertiary changes, such as the detachment of the inner and outer β-sheets and the translation of the C-loop observed with GLIC, are not resolved. Also, differences in the global twist and in the detailed mechanism of reorientation of TM helices are noted. Unfortunately, no molecular dynamics data are available on the recent GluCl data (Althoff et al. 2014).

The structure of a new “locally closed” (LC) state of GLIC—which shows a closed pore in a structure that preserves most of the features of the open form (Prevost et al. 2012)—also brings into question the simple correlation between global twisting and the tilting of the pore-lining helices, an issue thoroughly discussed in Sauguet et al. (2014a) and Cecchini and Changeux (2014).

Lastly, X-ray analysis of *Lymnaea* AChBP complexed with a series of 4,6-disubstituted 2-aminopyrimidines exhibiting both positive and negative cooperativity in ligand-binding assays shows that cooperative interactions are associated with a global* blooming* of the AChBP molecule, yet without a significant* twist* (Kaczanowska et al. 2014).

**Fig. 4:**
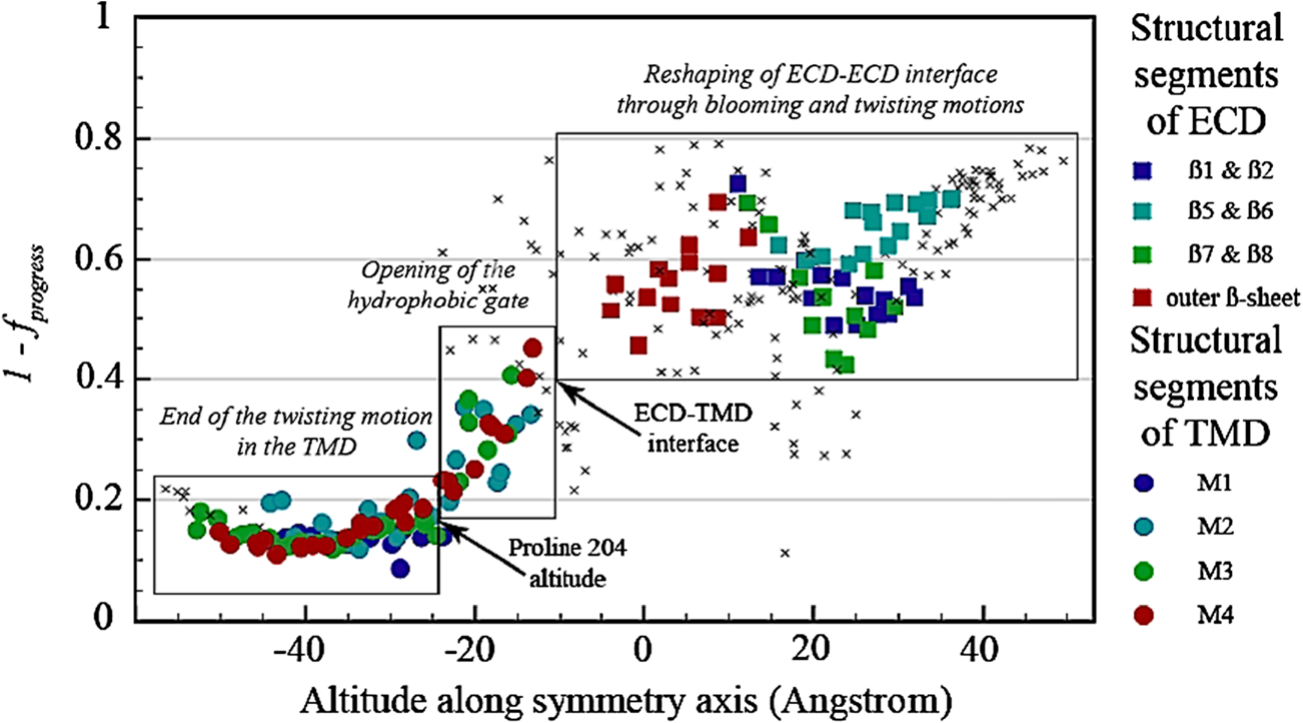
Coarsed grained molecular dynamics trajectories for the gating of the ion channel of the *G. violaceus* receptor. Opening of the ion channel is *from right to left*. The iENM web server (http://enm.lobos.nih.gov) was used to generate plausible trajectories between the four closed GLIC pH 7, the open GLIC pH 4 and the locally closed structures. The trajectories are mapped onto ECD and TMD reaction coordinates to quantify the differential progress of conformational change in these two domains. Residues belonging to structural segments (loops) are displayed with* colored symbols* (see color code on the* right*); all other residues are displayed with* small crosses*. The figure illustrates the stepwise progress of the concerted conformational change:* from right to left* channel opening process starting at the ECD,* from left to right* channel closing process starting at the TMD. From Sauguet et al. 2014a, Figure S18c.

Taken together, the most recent molecular dynamics (Calimet et al. 2013; Sauguet et al. 2014a; Taly et al. 2014; Cecchini and Changeux 2014), structural (Sauguet et al. 2014a) and physiological (Purohit et al. 2013) studies converge on a common atomic model of the gating transition (Sauguet et al. 2014a, b; Cecchini and Changeux 2014). According to the model, the “stepwise process” would start from the orthosteric-binding site (loops A, B and C), propagate to the EC/TM interface (β1– β2 loop and Cys loop) via a rigid-body rearrangement of the EC β-sandwiches and move down to the TM helices (first M2, then M4 and M3) to finally open the gate (Fig. 4). This process would involve two distinct sequential quaternary transitions: a radial concerted contraction or un-*blooming* of the EC domain, which promotes opening of the ion pore, followed by a global concerted *twisting* of the receptor to lock the channel in the active, open channel, state.

The data are consistent with single-channel recordings (Zheng and Auerbach 2011; Purohit et al. 2013) and their dynamics on a microsecond to millisecond timescale. They are also consistent with the MWC postulate that the conformational transition is “concerted” and, at the endpoints, conserves the symmetry of the oligomer. Furthermore, in agreement with the conformational selection mechanism, Sauguet et al. (2014a) observed that in some crystal forms of GLIC pH 4, the locally closed and open conformations are found to coexist as discrete entities. Interestingly, the greater fluctuations observed in crystals of GLIC pH 7 prefigured the conformational change toward the open state—as if the latter was already “on-path” even under conditions which did not favor its occurrence.

Finally, several fast and slow desensitized I and D states and uncoupled forms of prokaryotic receptors have been identified, and their structures are currently under investigation. For example, the locally closed state (Prevost et al. 2012) might be a fast desensitized I state (Prevost et al. 2013), and the closed state of ELIC—often considered to be a resting state (see Calimet et al. 2013)—might represent a slowly desensitized D one (Velisetty et al. 2012; Sauguet et al. 2014a). The ELIC structure has also been suggested to be in a “refractory” (Gonzalez-Gutierrez et al. 2012)/“uncoupled” state (daCosta and Baenziger 2013) (see Cecchini and Changeux 2014 for discussion).

## Allosteric modulation of receptor function: toward a new pharmacology

Several categories of allosteric sites modulate signal transduction mediated by nAChR. Ca^2+^, at millimolar concentrations, potentiates most neuronal nAChRs (Mulle et al. 1992; Vernino et al. 1992) and binds to sites located at the subunit interface of the EC domain, below the ACh site near the TM (Le Novere et al. 2002). In agreement with the MWC model, Ca^2+^ primarily affects the isomerization constant between the R and open A conformation (Galzi et al. 1996). A second class of allosteric sites lies within the TM and accommodates pharmacological agents which regulate receptor activity in either a positive (e.g. the antihelminthic ivermectin; Krause et al. 1998) or a negative [e.g. general anesthetics (GAs; for review, Nury et al. 2011) or ethanol (Sauguet et al. 2013)]. With mammalian GABA_A_ receptors, early studies by photoaffinity labeling with a derivative of the GA etomidate identified sites located both between and within subunits, in the TM domain (for review, Olsen 2014). Amazingly, GAs such as propofol and desflurane negatively modulate the response of the prokaryotic GLIC receptor. The X-ray structure of a GLIC–GA complex reveals that GAs bind to a common site within the upper part of the TM of each subunit inside a cavity accessible to phospholipids from the lipid bilayer (Nury et al. 2011). The entrance to this cavity is obstructed by a lipid alkyl chain that competes with propofol binding. Lipids, free fatty acids and steroids are known to allosterically modulate pLGICs and are thus likely candidates as endogenous ligands for the GA sites (Nury et al. 2011; Sauguet et al. 2013). Ivermectin, which positively modulates GluCl, binds to GluCl crystals at subunit interfaces between M3 and M1 (Hibbs and Gouaux 2011) and also competes with phospholipids (Althoff et al. 2014). It would appear that ivermectin is homologous to many important modulators of pentameric receptors, such as alcohols, anticonvulsants, anesthetics and diuretics (Sauguet et al. 2014b).

Molecular dynamic studies (Calimet et al. 2013; Sauguet et al. 2014a, b, Cecchini and Changeux 2014) of GluCl, and the crystal structures of GLIC pH 4 and pH 7 unambiguously show that the gating transitions involve a significant restructuring of the subunits’ interfaces. This restructuring includes a large contraction of the orthosteric sites in the R–A transition and a major change of the allosteric modulatory sites: widening of the homolog of the Ca^2+^ site pocket in the pH 7 R state and stabilization by positive allosteric modulators (ivermectin) of the untwisted (open pore) configuration of the TM site (Sauguet et al 2014a; Althoff et al 2014).

From a drug design perspective, the model suggests as logical targets the orthosteric and allosteric sites present on these defined conformation(s)—rather than a single rigid binding site (Changeux 2012a, 2013a; Sauguet et al. 2014b). It thus makes possible to selectively generate agonistic *versus* antagonistic-orthosteric or allosteric modulatory-ligands. The model also predicts that by altering the unliganded equilibrium between discrete conformational states, gene mutations may cause constitutive receptor activation (or inhibition) with important pathological consequences (Changeux and Edelstein 2005; Taly et al. 2006).

## Conclusions: receptor dynamics and higher brain functions

The introduction of molecular dynamics approaches in the analysis of the allosteric transition in pLGIC has led to a description, at the atomic level, of the conformational change mediating signal transduction and its intramolecular propagation as a concerted stepwise process at physiological timescales. It has also catalyzed understanding of the signal transduction mechanism mediated by ion channels (for review, Christopoulos et al. 2014) and other classes of membrane receptors, such as the G protein-coupled receptors (for review Bouvier 2013), the tyrosine kinase receptors or, even, the nuclear receptors (for review, Changeux 2012a, 2013b; Christopoulos et al. 2014).

The finding that some of the basic building blocks of the brain, such as the pentameric receptors, have not markedly changed for 3 billion years raises an interesting issue. The dynamics of our brain processes, including our mental ones, are constrained by the timescale of conformational transitions that originated in bacteria. This constraint possibly explains why our brain, which processes propagating signals below the speed of sound, looks so slow compared to computers operating at the speed of light.

These investigations further document and enrich what may be referred to as a “chemical theory of higher brain functions” (Changeux 1983, 2013b). Within this framework, would our ultimate mental processes rest upon the biochemical world of allosteric transitions that mediate interneuronal communications across the multiple levels of organization that span the human brain?
